# HIV/AIDS status disclosure increases support, behavioural change and, HIV prevention in the long term: a case for an Urban Clinic, Kampala, Uganda

**DOI:** 10.1186/1472-6963-14-276

**Published:** 2014-06-21

**Authors:** Lynn Muhimbuura Atuyambe, Eric Ssegujja, Sarah Ssali, Christopher Tumwine, Nicolate Nekesa, Annette Nannungi, Gery Ryan, Glenn Wagner

**Affiliations:** 1 Department of Community Health and Behavioral Sciences, Makerere University School of Public Health, P.O.Box 7072, Kampala, Uganda; 2 Research, Communication and Training (RECOT), P.O. Box 761, Kampala, Uganda; 3 Infectious Diseases Institute, Makerere University College of Health Sciences, Kampala, Uganda; 4 School of Women and Gender Studies, Makerere University, Kampala, Uganda; 5 RAND Corporation, 1776 Main Street, Santa Monica, California 90401-3208, USA; 6 Department of Health Policy, Planning and Management, Makerere University School of Public Health, P.O.Box 7072, Kampala, Uganda

**Keywords:** HIV/AIDS, Disclosure, Outcome, Uganda

## Abstract

**Background:**

Disclosure of HIV status supports risk reduction and facilitates access to prevention and care services, but can be inhibited by the fear of negative repercussions. We explored the short and long-term outcomes of disclosure among clients attending an urban HIV clinic in Uganda.

**Methods:**

Qualitative semi-structured interviews were administered to a purposeful sample of 40 adult HIV clients that was stratified by gender. The information elicited included their lived experiences and outcomes of disclosure in the short and long term. A text data management software (*ATLAS.ti*) was used for data analysis. Codes were exported to MS Excel and pivot tables, and code counts made to generate statistical data.

**Results:**

Of the 134 short-term responses elicited during the interview regarding disclosure events, most responses were supportive including encouragement, advice and support regarding HIV care and treatment. The results show on-disclosing to spouse, there was more trust, and use of condoms for HIV prevention. Only one third were negative responses, like emotional shock and feeling of distress. The negative reactions to the spouses included rejection, shock and distress in the short term. Even then, none of these events led to drastic change such as divorce. Other responses reflected HIV prevention and call for behavioural change and advice to change sexual behaviour, recipient seeking HIV testing or care. Women reported more responses of encouragement compared to men. Men reported more preventive behaviour compared to women. Of the 137 long-term outcomes elicited during disclosure, three quarters were positive followed by behavioral change and prevention, and then negative responses. Men reported increased care and support when they disclosed to fellow men compared to when women disclosed to women. There was better or not change in relationship when women disclosed to women than when women disclosed to men.

**Conclusions:**

There is overwhelming support to individuals that disclose their HIV status, especially in the long term. Besides, gender appears to influence responses to HIV disclosure, highlighting the need for gender specific disclosure support strategies.

## Background

Disclosure of HIV status remains a major hindrance in the fight against the spread of HIV in sub-Saharan Africa. Disclosing HIV test results to one's sexual partner allows the partner to engage in preventive behaviours, and the one who discloses can then better access the necessary support for coping with their HIV status and illness [1-3]. The positive attributes of disclosure include the ease to access HIV-Related services such as counselling and participating in education and training services [4], accessing ART services [5]. Furthermore, disclosure tremendously increases opportunities for obtaining social support, implementation of HIV risk reduction with partners and motivates the partners to seek voluntary counselling and testing (VCT) [6]. Disclosure may motivate partners to seek testing or reduce risk behaviour, and ultimately decrease the transmission of HIV [1]. Couples are encouraged to have their partners tested because of the expected benefits from institutions such as basic healthcare and material support from organizations [7], improved self-efficacy and communication [8]. A study done in Abidjan West Africa on Prevention of mother to child transmission of HIV (PMTCT) showed that there were benefits of HIV status disclosure as this led to more HIV testing among sexual partners [9]. It ought to be noted, however, that one of the stumbling blocks to disclosure currently is stigma as indicated in several studies [4,5,10].

Globally, research shows that gender plays an important role on whether or not to disclose ones HIV status. Females tend to experience more serious consequences of disclosure such as physical and sexual assault [11]. Furthermore, another study revealed that HIV infected women were two times less likely to disclose their status in fear of abuse, and divorce [12]. In terms of risk perception, women who perceived greater risk of HIV stigma, were less likely to disclose HIV status to spouse [13]. A study in Ethiopia demonstrated high disclosure rates to current partners for both males and females (90.9% vrs. 90.7% respectively) as it was customary to disclose ‘everything’ to ones partner. However, non-disclosure reasons varied by gender as men were concerned about their partner's worry and revelation of their own unfaithfulness. Conversely, the women feared physical violence, social and economic pressure in raising their children. The same study further showed that factors influencing disclosure were different by gender. Mens’ disclosure was motivated by the urge to know the partners HIV status and discussion prior seeking services while for women, the motivation was knowing the partner's HIV status and perceiving the current relationship as long-lasting [14]. This pool of evidence makes it difficult for women to disclose their HIV status and indeed this is an important factor in the HIV transmission dynamics.

Serostatus disclosure is an important component of secondary HIV prevention with potential benefits for both the individual and society (family and friends) by experiencing increased social support and by reducing HIV transmission risk behaviours respectively. Primarily, HIV disclosure takes place among sexual partners, family and friends. A study on safer sex behaviors in Kenya revealed for instance that unsafe sex was associated with lack of disclosure of HIV status to partner [15]. Another study of 73 HIV positive women who disclosed their serostatus to partners, family and friends, overall, few participants (less than 10%) experienced a feeling of regret after disclosure. Fifty nine percent of women experienced no regret (when they disclosed to all sexual partner 92%, all family 72%, all friend 79%) [16]. HIV disclosure to friends can result in either receipt of support (e.g., informational, emotional, material) and encouragement, or withdrawal, ridicule and even the end of a friendship. Disclosure to family involves risks such as negative emotional reactions, fear of stigmatizing children [7], fear of abandonment and fear of being disowned.

Research in Ethiopia revealed that among those who did not disclose their HIV status, 54% stated their reason as fear of negative reaction from their partner. However, among those disclosures, only 5% reported any negative reaction from the partner. The study concluded that although the majority of participants disclosed their test results, lack of disclosure by many resulted in a limited ability to engage in preventive behaviours and to access support. The study recommended that programmatic and counselling efforts should focus on mutual disclosure of HIV test results, by encouraging individuals to ask their partner's HIV status in addition to disclosing their own [1]. In another study among HIV-infected women who disclosed their HIV status, 82.1% declared that their partner had a “positive” reaction (was understanding and provided moral support). For the few women who declared “negative” reactions from their partner after disclosure, several were blamed for not discussing with them prior to HIV testing, one experienced violence, and a few ended relationship with their partner [9].

Research regarding disclosure to family and friends in sub-Saharan Africa is more limited, but also reveals both benefits and risks. Research in both high and low income countries revealed that in-spite-of the benefits such as preventive counselling (for safer sex practices) [17], disclosure makes it easier to ask and receive support from relatives and adult children once they know that there is a serious problem in the family. Little is known about whether immediate short term responses to disclosure remain stable or essentially unchanged such that responses several weeks or months after the disclosure events are very similar to the short term responses, or do responses to disclosure evolve over time as people adjust to the news that someone close to them is HIV-infected.

The main objective of this study was to describe the short (<3 months) and long-term (>3 months) outcomes when an HIV positive person discloses his/her status to different people such as spouse, family, friends and others. Our operational definition of short-term was a period not exceeding three months. This is the immediate stormy period when the partners experience highest physical, social and emotional reactions from each other and the immediate associates. This period does not usually last longer than three months. We also defined long-term as a period after three months of disclosure. This is the time when extra resources to cope (family, friends, workmates social institutions such as church) have been mobilized to offer support – essentially the stormy period will have calmed. The study addresses the following questions: When people living with HIV/AIDS (PLHAs) disclose their status, what responses do they receive in the short term? How do the responses or outcomes of disclosure to someone differ in the long term? How do the short and long term outcomes of HIV disclosure differ by gender, with regard to both the person disclosing and the recipient of disclosure?

## Methods

### Study design and setting

A qualitative study design was used with data collected from mid-February to mid-April 2008, using a semi-structured interview protocol at the Infectious Diseases Institute. This is a large HIV clinic in Kampala that operates as the national referral HIV/AIDS centre located within the Mulago National Referral Hospital Complex and has several specialized clinics that handle different complex HIV/AIDS co-morbidities. It also runs several satellite HIV clinics in the different districts within and outside Kampala the Capital city of Uganda.

### Selection of participants and sample

Participants were approached while waiting for their medical appointments, screened for eligibility, given information about the study and requested to participate. Those that agreed to participate first got their treatment from the provider and then were interviewed on exit. Four trained graduate research assistants obtained informed consent before proceeding with the interview. Patients that qualified for the interview were those above 18 years, clinically stable, and had enrolled for HIV care and treatment at the clinic for at least 6 months according to the study protocol.

The purposeful sample of 40 adult HIV clients was stratified by gender and age. This was intended to capture the different experiences that may be influenced by age and gender of the respondent. Participants included 10 younger men (age 18 to 34), 10 older men (age 35 and older), 10 younger women and 10 older women of similar age groups. Besides, this age cut off is the climax of HIV infection in the general population. Previous work elsewhere (Bishop et al. 2008) demonstrated that a sample of 40 respondents adequately captures the saturation levels required. Participants shared their retrospective disclosure experiences and the outcomes they received. On average, our participants had spent 5 to 6 years since being diagnosed with HIV, less than half (43%) had received any secondary school education, and most were working in the informal labour sector (87%).

### Interview protocol

The interview focused on eliciting information about direct disclosure, in which the person deliberately informed another of their HIV status, while non-disclosure referred to a deliberate decision not to disclose one’s HIV status. The targets of disclosure were disaggregated by social role and included spouse/partner, family members, friends, and others (for example workplace colleagues) in the community. Participants were asked if they had disclosed to someone in each of these target groups, and if so, to recount one disclosure event. Specifically, participants were asked: what exactly they had said or done to disclose their status and the short and long term (after three months) consequences or outcomes of the disclosure. Not all participants disclosed to all the social target groups. Where this was the case, participants were asked to provide their reasons for not disclosing to someone within the target group. The interviews were conducted by four graduate students and two senior research scientists, and in the language the participants were conversant with, but mostly English, Luganda and Runyankole/Rukiga. The interviews were audiotaped and then transcribed into English.

### Data analysis

Data related to the reasons for disclosure or nondisclosure have been published elsewhere [18]. For this paper, the analysis focused on the short and long term outcomes of disclosure. First, we used text management software (*ATLAS.ti*) to mark contiguous blocks of transcript text that pertained to the major topical domains of interest (how HIV status was disclosed, trigger event, reason for disclosure, short and long-term response to disclosure) within each social target group of the disclosure (spouse, family, friends, others). We then pulled out all text associated with a particular domain and after printing the quotes on slips of paper, the research team members sorted the quotes into piles based on their thematic similarities. Each thematic category that was identified was then given a name and an explicit codebook was developed detailing the inclusion and exclusion criteria for each category. We pulled the immediate outcomes and those that we deemed to be long term (over three months). In the next step, we matched each quote in a domain with a specific subcategory. We then conducted a code count for all the responses in a particular code categories that was used to examine the degree to which these themes were distributed across gender, age group, and social target group using percentages generated in MS Excel where pivot tables were generated.

We ensured reliability of our study instrument by pre-testing it and discussing pre-test results among the study team thereafter. Further to this, we performed an inter coder reliability test whereby the research team was divided into two teams to independently code eight transcripts of different categories of respondent. After that exercise these two teams came together and compared their code books and code definitions. A final harmonized codebook was then agreed upon and used to code our data.

### Ethical consideration

Informed written consent was obtained after the study was explained including potential risks and benefits, the voluntary nature of the study and ability to stop the interview at any time or not to answer specific questions. Standard precautions were undertaken to assure confidentiality of data; no identifying data were collected or documented aside from the consent form, which was kept in a locked cabinet, separate from the interview transcripts and data, and with access only to the study team members. All interviews were conducted in a private room within the HIV clinic. The study protocol was reviewed and approved by the Institutional Review Board at Makerere University School of Medicine and the Uganda National Council of Science and Technology.

## Results

### Main categories of short term disclosure outcomes

Participants described a total of 134 immediate or short-term responses to disclosure of their HIV status across all social target groups. When combining individual short-term response types into common themes, three main categories emerged: positive supportive responses, behavioural change and prevention responses, and negative responses. Table 1 below lists the range of short-term reactions to disclosure reported by the participants (Table 1).

**Table 1:** Range of short-term reactions after disclosure

**Short term outcomes to disclosure**	**Number**	**Percent**
**Negative**		
Negative emotional reaction	32	23.9
**Positive**		
Encouragement	36	26.9
Support	7	5.2
Did not change in relationship	27	20.1
**Prevention**		
Advice and support offered related to treatment and care	11	8.2
Advise on behavioural change	4	3.0
Recipient decided to get HIV tested	7	5.2
Recipient disclosed their HIV status	4	3.0
**Other**	6	4.5
**Total**	**134**	**100**

a) Positive and supportive responses to disclosure

While describing their experiences with disclosure, the respondents noted that they received positive and supportive messages from some of the people they disclosed their status to. During analysis, we identified responses that we considered to be positive support-related outcomes to disclosure. These included: encouragement and support, and no change in relationship. Hence, a total of 66 (59%) responses represented positive support. No change in relationship was categorized as a positive response because the person that has disclosed the status would keep enjoying the current relationship and support – i.e., business as usual.

The most common positive reaction was one of encouragement (27%). Other encouragement scenarios leaned towards adherence to HIV antiviral medication, and to live longer with a meaningful life. The quote below further demonstrates this view.

When the counsellor told me that I was infected, I got very angry. I went back home and told a friend about it. She comforted me and told me to remain strong and that I was not the first person to get infected. I also went and told my sister about it. She advised me to be strong and seek treatment. That is when I began coming to this clinic. (Young Female respondent)

The second main positive reaction when clients disclosed their HIV status was no change (17%) in relationship or relationship reportedly remained normal. This type of disclosure outcome was reported by both male and female participants. The following quotation captures this view;

Before disclosing, you first have to think a lot including the disclosure approach. It is the same thing that happened to me. I first thought a lot, and then later on I got the courage. … she also accepted. It appears she had already known the problem. The relation remained normal. It did not change. (Male respondent)

Positive responses that were supportive in nature were common in many of the responses. The support was in the form of finance and enhancement of relationship ties with the disclosure recipient. The quotes below demonstrate disclosure to a relative and the pledged support in the short term.

*Yes, except that she saw my skin and suggested that I should go, test and get to know my status. After testing, my sister told me not to fear of losing her because she emphasized that she likes me a lot.* (*Older woman disclosing to relative*)

At first, he was worried; he said ‘what shall we do’. I told him I do not know but they have told me of several treatment centres. He said, ‘that’s life, if it necessitates money, we shall get it for you to get treatment’. I was motivated by him to know that when I approach him for help, he will be willing to assist. (Older male disclosing to relative)

b) Behavioural change and HIV prevention responses

Our respondents mentioned receiving behavioural change and prevention responses when they disclosed their status. After the analysis process, the behavioural change and prevention category was comprised of the following short-term responses to disclosure: advised on behavioural change, advised on seeking treatment and care, the disclosure target decided to get tested for HIV, and target disclosed their HIV status. Over 19% of the disclosure responses reported by the participants reflected behavioural change and prevention. For example sexual partner’s accepted to use condoms after the participants had disclosed their HIV status. Receipt of advice on avoided sexual relations or involvement with the opposite sex, presumably to avoid transmitting the virus, was another type of response when participants disclosed. Advice on alcohol consumption and good nutrition was also reported by a few participants,*.* Regarding treatment and health seeking behaviour, there were a number of responses such as, “*She advised me to go to hospital and be helped*” *(Young male respondent)*, “*brother had promised to buy me ARVs but I told him I get them for free*”*(older male respondent)* and “*be strong and seek treatment, being positive doesn’t mean dying*”.*(older female respondent)*

c) Negative short term outcomes of HIV disclosure

During the disclosure process, respondents noted receiving negative outcomes in the short term. Two negative responses were encountered when respondents disclosed; (i) emotional reaction and (ii) rejection or outburst. About one quarter of the short-term responses to disclosure (24%) were negative emotional reactions. In these instances, those who were disclosed to often responded by expressing shock, being afraid, crying, and some made statements such as “my son you will die”, which reveals how many still view HIV disease as a ‘death sentence’. Results show that negative short term outcomes ranged from simply being upset to severe emotional reactions like rejection, angry outbursts and shock. For instance a young woman disclosing to her spouse that she was HIV-positive visibly saw ‘shock reaction’ from him.

I told him that I had gone for a test and had tested positive. I said this as I handed him the results. He was silent for some time and shook his head. He said it was very sad. I left him still seated and went to bed. He appeared more scared than I had been. (Young woman)

Rejection and outbursts were also commonly experienced in the short term after disclosure. Instances of couples separating beds and tones reflecting ‘not heeding to advice’ were common. Below are some of the examples.

*It was in the night when I disclosed to her. We had already finished eating supper and we had gone to bed. So I told her I have forgotten to swallow my medicine. ‘…give it to me so that I can swallow it’. So, she gave me the tin from which I got the tablets and swallowed them. Then I put back the tin onto the radio. At first I had feared to tell her. Then later on we started joking, and then I told her, you know what, the medicine that I am taking AIDS patients/AIDS patients*. *Then she got really annoyed, very annoyed. That night, she [left me in our marital bed and] slept on the floor. (Young man)*

### Do men and women differ by the types of short-term outcomes of HIV disclosure?

Using the themes derived we sought to understand whether there were differences and similarities between responses received by men and women when they disclosed their status. We observed that negative emotional reactions were equally received by both men and women with women receiving more encouragement responses compared with men.

Table 2 divides the 134 short-term outcome responses reported by the sample into those reported by male participants and those reported by female participants. For example, of the 68 responses that the male participants reported, 17 (25%) of them were negative emotional reactions. Similarly of the 66 responses the female respondent’s received 15 (23%) were negative emotional reactions (Table 2). Disclosure also led to quarrels in families and at times led to a state of hopelessness.

**Table 2:** Prevalence of short-term outcomes by participant’s gender

**Short term outcomes of disclosure**	**Male**		**Female**		**Total**
	**n**	**%**	**n**	**%**	**N**
**Negative**					
Negative emotional reactions	17	25	15	22.7	32
**Positive**					
Encouragement	15	22.1	21	31.8	36
Support	3	4.4	4	6.1	7
Did not change in relationship	11	16.2	16	24.2	27
**Prevention**					
Advice and support offered related to treatment and care	7	10.3	4	6.1	11
Advised on behavioural change	3	4.4	1	1.5	4
Responded with regard to target getting tested	5	7.4	2	3	7
Disclosure	3	4.4	1	1.5	4
**Other**	4	5.9	2	3	6
**Total**	**68**		**66**		**134**

I told him that I am positive and … he said why can’t you go to the village and they give you your property and sell it? Do you want to leave your wealth behind to be consumed by other people? (Male respondent)

On the other hand, short-term outcomes from recipients reflecting ‘encouragement’ were reported somewhat more by females (32%) than males (22%). For example one of the male participants who received this kind of response had this to say:

I didn’t meander when I was disclosing to him, I just disclosed to him direct… He told me that ‘Jacobin (not real name) you did a good thing to go and test. You would have got a big problem but now that you have known that you have HIV and the Doctors are giving you treatment, that will help you. If you had delayed to go for testing, the disease would have matured without you knowing’. (Old male respondent)

### Influence of disclosure recipients’ gender on the type of responses received by PLHAs

Among the factors that influenced the disclosure outcomes was the gender of the recipients of disclosure. Regarding positive support outcomes of disclosure, slightly more female recipients responded to disclosure with positive support compared to men (67% versus 54%). In addition, our data further revealed that when females disclosed to males, they mostly received positive responses that included support and no change in relationship (65%), which is only slightly more than when females disclose to other women (58%). When males disclosed to males, two thirds (67%) of the reported outcomes were positive, whereas the proportion of positive responses from female recipients reported by men was a much higher 84%. Furthermore, with regard to receipt of encouragement, nearly half (46%) of the outcomes to disclosure reported by women were characterized as reflecting encouragement when other females were the recipient, compared to only 27% of the outcomes reported by men where males were the recipient. The experience of negative emotional reactions to disclosure were similar across male (21%) and female (22%) recipients. Regarding prevention and behavioral change, it is the males who were more likely to receive it (14%) than females (9%) (Table 3).

**Table 3:** Proportion of short-term broad categories of responses by gender of recipient

**Short term outcomes of disclosure**	**Male**		**Female**	
	**n**	**%**	**n**	**%**
Positive	37	56.9	44.0	63.8
Prevention and behaviour change	9	13.8	6.0	8.7
Negative	17	26.2	15.0	21.7
Other	2	3.1	4.0	5.8
Total	65		69	

Differences were registered when participants disclosed to the opposite sex. For example, while disclosing to females, males received negative emotional reactions (33%) as opposed to when they disclosed to fellow males (13%). Below are quotations from a respondent that reflect this;

Immediately I knew like this, I went back home and I told them that ‘you people, they have tested me and found that I am positive’. So my brother just told me that fine. If that’s the case, let them write you the medicine and I will buy it’. (male respondent disclosing to male)

I disclosed to her because she is also born again and we used to pray together. She would come to me every Sunday morning and asks me to go to church…. She was terrified and cried…she asked me that ‘where did you get it from’. … you know women, for her she got so scared (male respondent disclosing to female)

### Long term outcomes of HIV disclosure

Disclosure outcomes stretched from the immediate/short-term outcomes into the long term outcomes. These were mainly the experiences they received three months onwards from the time they disclosed their status. HIV positive clients were asked to give the long term outcomes when they disclosed their HIV status to different recipients (spouse, children, family, friends and others). These were related to the same disclosure events discussed in the short term outcomes. Table 4 shows that after some time has passed following the disclosure event (i.e. after 3 months), the long term outcomes were mainly positive.

**Table 4:** Range of Long term reactions after disclosure

**Long term outcomes of disclosure**	**Number**	**Percent**
Did not change	36	26.3
Relationship is stronger/increased caring and support	39	28.5
Given care and treatment	10	7.3
Given material support	11	8.0
Improved work conditions	4	2.9
Behavioural change and positive living (advice and practice)	12	8.8
Prevention advocacy effects on others	9	6.6
Negative outcomes	13	9.5
Other	3	2.2
**Total**	**137**	**100**

### Categories of long term disclosure outcomes

Like the short term outcomes, the three main categories of the outcomes in the long run were positive response, behavioural change and prevention, and negative responses. The overall majority of the responses were positive (73%), these were followed by the behavioural change and prevention (15.3%), negative responses (9.5%) and other (2.2%).

a) Positive disclosure outcomes

The positive responses reflected were in terms of encouragement and support, adherence to medication, and good nutrition. One of the participants pointed out how she started sharing medical information and assistance in keeping clinic appointments with a fellow patient at the clinic who became aware of her HIV status.

The first time we met at the clinic, He just told me that he had been here for quite some time and left it at that. Later we began sharing ideas on how we would be reminding each other about the appointments and about the time for taking medicine. Isn’t this wonderful! (Female respondent)

For me my friend reminds me to take my drugs when I have delayed. He has also been reminding me of my appointments. (Male respondent)

Food and nutrition was recognized by recipients as important for recovery and wellness, as illustrated by one female recipient.

Months after disclosure, she became very caring. She would give me fruits and money to buy fish so as to restore my health just like any other person would care for their sick ones. (Female respondent).

In some instances, trust was enhanced in the relationship after the disclosure. There was more openness in financial matters and delegation of activities involving monitory transactions.

He trusts me very much, even with money. He is happier that I resumed work, he even trusts me more! Our relationship has even grown stronger than before. (Female respondent)

b) Long term behaviour change and prevention messages

The second broad category of long term outcomes of disclosure was ‘behaviour change and prevention messages’. The main HIV behaviour change prevention messages focused on adjustments in the sexual behaviour and other HIV preventive behaviours. Specifically, condom use was highlighted as one of the messages deemed critical for HIV prevention. In some cases disclosure in the long run facilitated planning for safe child delivery, especially for participants that still wanted to have children. One such couple enrolled in a prevention of mother-to-child-transmission (PMTCT) program following the disclosure, “*You know since disclosing to my wife, she decided to go for the PMTCT program because she wanted to have children” (male respondent).*

A number of participants indicated advising the recipient to take an HIV test as a prevention strategy. This was mainly to the children and close relatives whom they had social influence on.

On realizing that people were positive, I have tested my children and they are all negative. I also advised my niece to test, so she went and tested. (Female respondent)

My girlfriend cautions me not to engage in other relationships because I can bring other strains of HIV. (Male respondent)

c) Negative Long term outcomes of disclosure

The negative responses that people received in the long run after they disclosed were mostly of the emotional nature, evoking bad memories of abandonment and ‘coming to the realities of being HIV positive. These continued happening even after some time had passed from the time of the disclosure event. Statements such as “*He abandoned me in his house, claiming I have another husband; he went forever” (older female respondent),“She sometimes quarrels, sometimes she is okay, but not as loving as before”(older male respondent) and “His boss has continued stigmatizing him*”*(older male respondent)* were common within the negative long term responses to disclosure. One respondent noted that his girlfriend “*gets depressed whenever we discuss our HIV positive status” (older male respondent)”,* while another noticed that her partner seems to “*no longer care much about me*”.*(older female respondent after disclosing to husband)* These findings reflected that outcomes of an emotional nature took time to fade away and continued to be experienced in the long-term.

Table 5 shows that with time after disclosing one’s HIV status, participants experience different outcomes. Overall, the outcomes of disclosure were positive, though to varying degrees according to gender. For example men experienced stronger relationship and increase in care and support (33%). Similar to the short terms outcomes, men (14%) received more advice related to behaviour change and positive living than women (2%). It should be noticed that when men disclose their HIV status to others, the recipients tend to respond more with prevention advocacy messages than when women are disclosing their status (10% vs. 2%).

**Table 5:** Long term outcomes of disclosure by gender of participants

**Long term outcomes of disclosure**	**Male**		**Female**		**Total**	
	**N**	**%**	**n**	**%**	**N**	**%**
Did not change	9	11.5	27	45.8	36	26.3
Relationship is stronger/increased caring and support	26	33.3	13	22.0	39	28.5
Given care and treatment	7	9.0	3	5.1	10	7.3
Given material support	5	6.4	6	10.2	11	8.0
Improved work conditions	4	5.1	0		4	2.9
Behavioural change and positive living (advice and practice)	11	14.1	1	1.7	12	8.8
Prevention advocacy effects on others	8	10.3	1	1.7	9	6.6
Negative outcomes	6	7.7	7	11.9	13	9.5
Other	2	2.6	1	1.7	3	2.19
**Total**	**78**		**59**		**137**	**100**

Qualitative data also affirmed this view

She used to love me because she is my mother and we had to get on well such that whenever I had difficulties I would go to her and she helps me. In case she failed to help, I would go to my family members and tell them about the issue… She didn’t change. She remained the same and always consoled me… of course previously when my husband was alive she would care but not so much because she knew I had a man who would care for me. But nowadays whenever she gets to know that I have a problem, she tries to solve it. (Female respondent)

### Proportion of type of responses by gender of recipients

#### Influence of recipients’ gender on the long term outcomes of HIV disclosure

Like in the short term, the recipient’s gender had an influence on the type of responses that respondents reported receiving. Results revealed that when males disclosed to males, their relationship often became stronger or received increased care and support (50%). On the other hand only one quarter of responses show that when males disclosed to females their relationship became stronger or received increased support (27%). Results further reveal that there was a 50% chance of no change in the relationship with the recipient when males disclose to males as opposed to when males disclosed to females (33%).

… *I don’t have any problems with him and he takes it like I am not even infected because I go to his place, then we go to church and pray together. I think it’s good because if I kept quite I wouldn’t have got the advice that he gave me like giving up on girls. I used to like booze but he told me that I should stop it. (Young male respondent)*

For females disclosing to fellow females, they report mainly a no change in the relationship (52%), whereas when women disclose to males only a third (33%) reported no change to relationship outcomes, while another third (33%) reported experiencing increased care and support in the relationship. The vivid typical quotes follow:

…but my other siblings are there in Masaka far away from here. They cannot even help me quickly because of distance. Even then still, it is this one that I trust. There has been no difference in relationship since I told her, no misunderstandings at all so far. (Older female respondent)

We talked about it again. He told me that I would be fine once I start taking ARVS. I took the medicine for about two months without any change then he asked me why there was no change yet I was taking the medicine. During the third month I began noticing changes…. We remained like we used to be before. (Young female respondent).

#### Comparison between the short and term outcomes of disclosure

We sought to use the data to observe if there were variations between the short term and long term outcomes of disclosure. Table 6 suggests that after disclosure of HIV status the benefits increase over time. For example the amount of positive outcomes of disclosure increases from 60.4% in the short term to 73% in the long term. The same trend was observed for prevention and behaviour change where it increased from 11.2% to 15.4%. On the other hand there was a reduction in the negative outcomes of disclosure from 23.9% in the short term to 9.5% in the long term. Among the 32 recipients of disclosure who responded negatively in the short-term, 13 were still responding negatively in the long-term compared to 19 whose response had evolved into a positive support response. Of the recipients that remained negative in the long term 6 were males while 7 were females.

**Table 6:** Proportion of long term broad categories of responses by gender of recipient

**Long term outcomes of disclosure**	**Male**		**Female**	
	**n**	**%**	**n**	**%**
Positive	51	65.4	49	83.1
Prevention and behaviour change	19	24.4	2	3.4
Negative	6	7.7	7	11.9
Other	2	2.6	1	1.7
	**78**		**59**	

## Discussion

This exploratory work highlights key outcomes of disclosure of one’s HIV status both in the short and long-term period (three months and above) as was operationally defined. In the short and long-term, when individuals disclose their HIV status, they appear to receive mostly positive reactions from those they chose to disclose to such as family, friends, and workmates. This is an important finding because one of the reasons people do not disclose is fear of disclosure repercussions such as violence, separations and withdrawal of support, and negative emotional reactions [2,19]. HIV disclosure is important for critical public health benefits such as HIV prevention advocacy, HIV testing, protection from infection, and early enrolment on ART [2,3] and adherence to the medications — all of which are supported by the responses to disclosure described in this study.

Our data also revealed that when HIV positive people disclosed their serostatus, the short-term reactions were often of ‘behaviour change and HIV prevention’ in nature. Advise on seeking treatment and care, and HIV prevention such as consistent condom use are prominent. One of the reasons HIV incidence has not receded in Uganda is because of the new infections which are attributed in part to infected individuals who have not been HIV-tested unknowingly infecting others [20]. The Uganda Health Sector Strategic and Investment Plan prioritizes the prevention and control of HIV/AIDS, with increased focus on HIV prevention among couples and other high risk groups such as commercial sex workers [21]. It is, therefore, important to ramp up strategies to increase HIV disclosure and HIV testing. Promoting the “positive prevention”, whereby HIV-infected persons are encouraged to reduce HIV risk behaviour, is also key to HIV prevention as PLHA who do not know their HIV status could knowingly or unknowingly spread the infection. Disclosure helps to facilitate the uptake of HIV testing among recipients of the disclosure.

About one quarter of the short-term outcomes of disclosure were negative (mainly emotional reactions such as shock, crying, feeling afraid). Besides being a relatively small percent, this negative type of reaction is not strange in the short term, given the nature of the disease (long illness, no cure, lifelong medication, and expected high morbidity). This optimistic finding could imply that the public ought to be encouraged to disclose their HIV status to close networks as they look forward to reap disclosure benefits. We note in our findings that recipients that responded with negative outcomes in the short term gradually changed to give positive outcomes in the long term. However, some of the recipients that gave negative outcomes in the short term remained negative in the long term. This category did not differ by gender. This implies that the immediate negative reactions could be the shock which after all normalizes or even becomes positive.

In terms of gender, our study revealed that the females tend to receive more sympathy and encouragement than males probably because of the gender dynamics and norms in the sub-Saharan region. Society especially in Africa tends to ascribe men to be strong, courageous, fearless and are therefore expected to cope better than women especially in difficult times. However, it is important to note that this may not always be the case especially for those who do not have positive coping resources [22]. Failure to cope could also lead to negative outcomes such as denial on the part of men. This, therefore, implies that that programs supporting men need to be established or strengthened where they exist.

Our results revealed that long-term outcomes of disclosure were overwhelmingly positive, with even fewer negative outcomes in the long term (9.5) compared to the short term responses. This reconfirms and assures the HIV positive individuals that when they disclose, the risks are indeed much fewer in the long term. The public health benefits of disclosure such as HIV testing and safer safe practices [17], support to spouse (emotional, financial, material, and a treatment supporter for the life- long ART) [18], increased self-efficacy (feeling confident telling someone about HIV status, feeling certain on deciding to disclose) over time [8] have been documented in other settings in the Sub-Saharan region as well. A study in Abidjan revealed that among HIV-infected women who disclosed their HIV status, 82.1% declared that their partner had a “positive” reaction, i.e., was understanding and provided moral support. Among the women declaring “negative” reactions from their partner after disclosure, only 10 (4%) were blamed for not discussing with the partner prior to HIV testing. Further still, only one (0.4%) experienced violence, and six (2.4%) ended their relationship with their partner [9]. Yet in another study in Uganda, disclosure of HIV status was very advantageous. Overall, 80% of the women had disclosed their HIV status with most reporting positive outcomes. They developed ‘adaptive coping strategies’. Besides, this study also revealed that HIV serostatus disclosure was closely related to the development of support networks that helped women to come to terms with the their diagnosis [23]. Indeed this confirms that disclosure has overwhelming benefits and should be encouraged as much as possible.

When we compared the short and long term outcomes of disclosure, the results revealed that as HIV positive people in care disclose their status in the short-term, the amount of positive outcomes plus prevention and behaviour change outcomes continue to increase in the long term. On the other hand the negative outcomes received in the short term drastically reduce. This implies that the myths and fears of HIV disclosure and the consequences are real and realized by some people. However our data suggests that people respond more favourably to disclosure than perhaps expected. This has an important public health message.

Finally, we note that our study has a limitation that our data gives a cross-sectional picture of the short and long term outcomes of disclosure. We did not track individual’s response to disclosure over time. Therefore we cannot address the change over time question of individuals’ responses.

Similarly, there is a possibility of recall bias. Even though the diagnosis of HIV was about 5 to 6 years back, disclosure could have been more recent, thereby minimizing this bias. Besides, we believe that participants describing their life time lived experiences of disclosure often is hard to forget, therefore recall bias was minimal.

## Conclusions

Our compelling results suggest that when HIV positive individuals disclose their serostatus, they commonly receive positive and supportive responses. This is contrary to the popular view that recipients of disclosure news react negatively. There is therefore a strong need to promote realistic and effective HIV disclosure decision making in order to help realize the public health and personal benefits of disclosure. The main media (electronic, print) need to take on this important role.

## Competing interests

The authors declare that they have no competing interests.

## Authors’ contributions

LA, GW, SS, GR contributed to the study concept and design, data collection and supervision, analysis and write up of the manuscript. ES, CT, NN, AN, contributed to the data collection, analysis and commenting on the manuscript. All authors read and approved the final manuscript.

## Pre-publication history

The pre-publication history for this paper can be accessed here:

http://www.biomedcentral.com/1472-6963/14/276/prepub
